# Exploring the association of staff characteristics with staff perceptions of quality of life of individuals with intellectual disabilities and challenging behaviours

**DOI:** 10.1111/jar.13017

**Published:** 2022-06-10

**Authors:** Eke Bruinsma, Barbara J. van den Hoofdakker, Pieter J. Hoekstra, Gerda M. de Kuijper, Annelies A. de Bildt

**Affiliations:** ^1^ Department of Child and Adolescent Psychiatry University of Groningen, University Medical Centre Groningen Groningen The Netherlands; ^2^ Department of Clinical Psychology and Experimental Psychopathology University of Groningen Groningen The Netherlands; ^3^ Centre for Intellectual Disability and Mental Health Assen The Netherlands

**Keywords:** challenging behaviours, intellectual disabilities, quality of life, staff characteristics, team characteristics

## Abstract

**Background:**

This study aimed to examine the associations between individual staff and staff team characteristics and quality of life of individuals with intellectual disabilities and challenging behaviours.

**Method:**

With multilevel analyses, we examined educational level, experience, attitudes and behaviours of 240 staff members, in relation to their perception of quality of life of 152 individuals with intellectual disabilities and challenging behaviours they cared for.

**Results:**

Two individual staff characteristics were related to better quality of life: higher educational and self‐reflection levels. Of the team characteristics, higher educational level, higher self‐efficacy and more friendly behaviour were associated with better quality of life. Unexpectedly, higher staff‐individual ratio was related to lower quality of life.

**Conclusions:**

Both individual staff and staff team characteristics are associated with quality of life, indicating the need to take staff team characteristics into account when examining quality of life.

## 1 INTRODUCTION

Individuals with intellectual disabilities and challenging behaviours, also defined as ‘behaviours of concern’ (e.g., Nankervis & Chan, 2021), are at risk of experiencing a lower quality of life than individuals who do not display these behaviours (Simões & Santos, 2017). They more often experience physical injury, social exclusion and lack of autonomy (Allen et al., 2009; Griffith et al., 2013; Matson & Boisjoli, 2009; Sturmey et al., 2005). These are violations of several domains of quality of life as described in the model of Schalock and Verdugo (2002) that distinguishes eight core domains of quality of life: (1) personal development; (2) self‐determination; (3) interpersonal relations; (4) social inclusion; (5) rights; (6) emotional well‐being; (7) physical well‐being; and (8) material well‐being. According to this model, quality of life is a multidimensional framework that is influenced by the individual themselves (micro‐level), as well as their immediate environment (meso‐level) and the society they life in (macro‐level).

In residential care settings, the immediate environment consists of the direct staff members, who play an important role in the quality of life of the individuals under their care (Claes et al., 2012; Jenaro et al., 2013; Rose, 2011). However, it is unclear how staff characteristics such as age, sex, education level and work experience, but also their attitudes or reactions towards challenging behaviours, are related to the quality of life of the individuals they care for. Knowledge on the relationship between staff team characteristics (e.g., the number of experienced staff members within a team) and quality of life is even more scarce. More insight into the role of staff may provide directions for improving quality of life of individuals with intellectual disabilities and challenging behaviours who live in residential care settings.

The limited quantitative research on the relationship between staff and quality of life of individuals with intellectual disabilities and challenging behaviours has mostly focused on specific domains or indicators of quality of life, such as social inclusion (Bigby & Beadle‐Brown, 2018; Claes et al., 2012; Felce et al., 2002a; McConkey & Collins, 2010; Perry & Felce, 2003), emotional well‐being (Sexton et al., 2016) or self‐determination (Rossow‐Kimball & Goodwin, 2009; Stancliffe, 2001; Wehmeyer & Bolding, 2001). Staff characteristics that have been studied in relation to social inclusion are sex, educational level, work experience and staff‐individual ratio. Male staff members more often considered stimulating social inclusion of the individuals under their care as part of their job and prioritised it more often than female staff (McConkey & Collins, 2010). Some studies found that higher educational level of staff was related to more social inclusion and self‐determination (Mansell et al., 2008; Thomas et al., 1978), whereas in other studies these relationships were not confirmed (Felce et al., 2002a, 2002b). With regard to work experience, it has been demonstrated that more experienced staff initiated more social activities with the individuals under their care (Mansell et al., 2003), however, they also used more physical restraint (Emerson et al., 2000). Regarding team characteristics, only a very limited number of studies have been conducted, indicating that a higher staff‐individual ratio did not enhance quality of life (Beadle‐Brown et al., 2016; Felce et al., 2002a). It is important to further investigate team characteristics, since individuals with intellectual disabilities are being cared for by staff teams rather than individual staff members. Characteristics of one single staff member may have a smaller impact on the quality of life than the characteristics of a complete team.

Some qualitative studies have provided valuable suggestions for desirable staff characteristics that may enhance quality of life, based on the views of individuals with intellectual disabilities, staff, and family members (Frounfelker & Bartone, 2020; Petry et al., 2007; Webb et al., 2020; Windley & Chapman, 2010). All respondents considered work experience, sensitivity, self‐reflection, friendly behaviour, knowledge, patience, and being caring and empathetic to be important staff characteristics (Petry et al., 2007; Webb et al., 2020; Windley & Chapman, 2010). Hostile staff behaviour, on the other hand, was mentioned as a factor that was expected to decrease quality of life (i.e., lower emotional well‐being, less self‐determination; Griffith et al., 2013). Furthermore, staff members stated that they need more training on human rights to improve the quality of life of the individuals under their care (Windley & Chapman, 2010), indicating a need for knowledge.

Aside from the few quantitative studies examining the direct relation between staff factors and quality of life, other studies have investigated staff characteristics that may be more indirectly related to quality of life. For example, it was demonstrated that working experience, self‐efficacy, attributions of staff about the controllability of challenging behaviours, positive emotions of staff when confronted with challenging behaviours (Willems et al., 2016), and male gender of staff (Willems et al., 2014) were related to more friendly staff behaviour, which has been hypothesised to enhance quality of life (Webb et al., 2020). Moreover, negative staff emotions as a reaction to challenging behaviours were associated with hostile behaviour of staff (Willems et al., 2016), which was mentioned as a possible factor to negatively impact quality of life (Griffith et al., 2013). Self‐reflection, higher educational level, proactive thinking and support‐seeking behaviour were related to less frequent hostile staff behaviour (Willems et al., 2010, 2016).

The aim of this study was to expand the body of research on the associations between staff characteristics and quality of life of individuals with intellectual disabilities and challenging behaviours in two ways. First, we examined the relation of individual staff characteristics (i.e., sex, experience, friendly behaviour, positive and negative emotions to challenging behaviours, attributions about control, self‐efficacy, educational level, self‐reflection, proactive thinking, assertive control and hostile behaviour) and staff team characteristics (i.e., sex, experience, friendly behaviour, positive and negative emotions to challenging behaviours, attributions about control, self‐efficacy, educational level, self‐reflection, proactive thinking, assertive control and hostile behaviour) with quality of life of individuals with intellectual disabilities and challenging behaviours in group homes in the Netherlands. We expected male staff, more working experience, friendly behaviour, positive emotions in response to challenging behaviours, belief in external controllability of challenging behaviours, higher self‐efficacy, higher educational level, higher self‐reflection, more support seeking‐behaviour, and more proactive thinking of individual staff or teams to be positively associated with quality of life. Furthermore, we hypothesised that negative emotions in response to challenging behaviours, more hostile behaviours, more assertive control, and more critical expressed emotions of staff or teams to be negatively associated with quality of life.

Second, to assess quality of life, we investigated total quality of life (i.e., the sum of all eight domains) and, building upon previous research, three specific domains: self‐determination (Rossow‐Kimball & Goodwin, 2009; Stancliffe, 2001; Wehmeyer & Bolding, 2001), social inclusion (Bigby & Beadle‐Brown, 2018; Claes et al., 2012; Felce et al., 2002a; McConkey & Collins, 2010; Perry & Felce, 2003) and emotional well‐being (Sexton et al., 2016).

## 2 METHOD

### 2.1 Study design

The present study was part of a multi‐centre cluster controlled trial, examining the efficacy of a Positive Behaviour Support training for staff in the Netherlands. The trial included four assessments (one pre‐test, one mid‐test and two post‐tests). Data from the pre‐test were used for the purpose of this study, resulting in a cross‐sectional design.

The Medical Ethical Committee of the University Medical Centre Groningen judged that the Medical Research Involving Human Subjects Act (WMO) did not apply to our study. We conducted the study in accordance with the World Medical Association (WMA) Declaration of Helsinki (2013).

### 2.2 Participants

In the Netherlands, each group home for individuals with intellectual disabilities and challenging behaviour has its own specific team of staff providing care for its residents. For our study, we included complete teams of staff and the individuals with intellectual disabilities and challenging behaviours they cared for. The teams were from different organisations in the Netherlands. The individuals the teams cared for and reported on had to meet the following criteria: (1) the individuals were 18 years or older; (2) the individuals had a mild, moderate or severe intellectual disability; (3) the individuals lived in a group home and received 24 h care each day; (4) the individuals interacted with staff at least 2 h a day; (5) the individuals displayed challenging behaviours at least once a week, as assessed by the staff members with the Aberrant Behaviour Checklist (ABC; Aman et al., 1985).

### 2.3 Procedure

All organisations that were member of the Dutch Association for Care for Individuals with Intellectual disabilities (in Dutch ‘Vereniging Gehandicaptenzorg Nederland; VGN’) were contacted for participation. Team managers and/or psychologists of interested organisations selected eligible teams and group homes. Meetings in which information was provided about the research project were held for teams that were interested. When staff members consented to participate, similar informative meetings were organised for legal guardians of the individuals with intellectual disabilities. After that, staff contacted the legal representatives for consent for using data about the individual concerned. For every individual for whom consent was given, a staff member was selected by the psychologist as informant. In the majority of cases, this was the primary staff member (i.e., the staff member responsible for contact with the family and adherence to the treatment plan), alternatively another staff member who knew the individual well. Almost all informants were appointed to one individual with intellectual disabilities and challenging behaviours to conduct the assessments. Three informants completed assessments on multiple (i.e., three) individuals with intellectual disabilities and challenging behaviours.

Assessments consisted of three components: (1) a 30 min online questionnaire for all staff members, including questions about demographic variables, interpersonal and intrapersonal behaviour of staff towards individuals displaying challenging behaviours, causal attributions about challenging behaviours, emotional reactions to challenging behaviours and self‐efficacy in working with individuals with challenging behaviours; (2) a 10 min online questionnaire for the primary staff member about demographics, recently displayed challenging behaviours and the current behavioural functioning of the individual; and (3) a telephone interview with the primary staff member on the quality of life of the individual with intellectual disability and challenging behaviours. The questionnaires were conducted through the online survey software Qualtrics (Qualtrics, Provo, UT). All interviews were carried out by the first author.

### 2.4 Measures and instruments

#### 2.4.1 Demographics staff and individuals with intellectual disabilities and challenging behaviours

Information about age, sex, work experience and staff team sizes was collected from all the participating staff members. Regarding the individuals with intellectual disabilities and challenging behaviours, we collected specific demographics (i.e., age, sex, work experience and staff team size) with the help of the primary staff member.

#### 2.4.2 Staff attributions: External control of the causal dimension scale

Attributions of staff about the degree to which the environment has control over challenging behaviours was measured with the subscale External Control of the Causal Dimension Scale (CDS‐II; McAuley et al., 1992). This subscale consists of three 9‐point Likert items ranging from 1 (cannot regulate) to 9 (can regulate). An example of an item is: ‘Is the cause of challenging behaviour something other people can or cannot regulate?’. The CDS‐II has good internal consistency (*a =* 0.82) and sufficient construct validity (McAuley et al., 1992).

#### 2.4.3 Staff self‐efficacy: Challenging Behaviour Self‐Efficacy Scale

To determine self‐efficacy in staff in dealing with challenging behaviours, we used the Challenging Behaviour Self‐Efficacy Scale (CBSES; Hastings & Brown, 2002). The CBSES consists of five items covering the following concepts: (1) confidence; (2) control; (3) satisfaction in dealing with challenging behaviours; (4) perception of a positive impact on the challenging behaviours; and (5) difficulty working with challenging behaviours. Each item has to be rated on a 7‐point Likert scale ranging from 1 (not at all confident) to 7 (very confident). The Dutch version of the CBSES has a good level of internal consistency (*a* = 0.85; Willems et al., 2016).

#### 2.4.4 Emotional reactions of staff: Emotional Reaction to Challenging Behaviour Scale

Positive emotional reactions of staff to challenging behaviours were assessed with the Emotional Reaction to Challenging Behaviour Scale (ERCB; Jones & Hastings, 2003). This 23‐item scale comprises a list of positive (e.g., confident, relaxed, cheerful and excited) and negative (e.g., depression, anger, fear and anxiety) emotions that are potentially experienced by caregivers when working with individuals who display challenging behaviours. Each item has to be rated on a 4‐point Likert scale ranging from 0 (no, never) to 3 (yes, almost always). The internal consistency of the original questionnaire and of the Dutch translation are good (*a* ranges: 0.69–0.86 and 0.82–0.84, respectively; Willems et al., 2016).

#### 2.4.5 Interpersonal and intrapersonal staff behaviour: Staff‐Client Interactive Behaviour Inventory

Interpersonal and intrapersonal behaviour of staff was measured with the Staff‐Client Interactive Behaviour Inventory (SCIBI; Willems et al., 2010). This questionnaire distinguishes four subscales of interpersonal staff behaviours: Assertive Control, Hostile Behaviour, Friendly Behaviour and Support‐Seeking Behaviour; and three subscales of intrapersonal staff behaviours: Proactive Thinking, Self‐Reflection and Critical Expressed Emotion. The SCIBI consists of 30 items that have to be scored on a 5‐point Likert scale ranging from 1 (completely inapplicable) to 5 (completely applicable). An example of an intrapersonal item is: ‘In working with this client, I have the tendency to approach him cynically’. The internal consistency of the SCIBI is good (*a* range: 0.70–0.89; Willems et al., 2010).

#### 2.4.6 Quality of life domains: Personal Outcome Scale

To assess self‐determination, social inclusion, emotional well‐being and quality of life of the individuals with intellectual disabilities and challenging behaviours we administered the Personal Outcome Scale (POS; van Loon et al., 2013) to the informant of each individual. The POS measures quality of life of people with intellectual disabilities and includes eight subscales, corresponding with the eight domains of quality of life as distinguished by Schalock and Verdugo (2002): Personal Development, Self‐Determination, Interpersonal Relations, Social Inclusion, Rights, Emotional Well‐Being, Physical Well‐Being and Material Well‐Being. Every subscale includes six items on a 3‐point Likert scale ranging from 1 (never) to 3 (always), resulting in a minimum total score of 6 and a maximum of 18. An example of a Self‐Determination item is: ‘Does the person have enough money to make choices?’, and an example of an Emotional Well‐being item: ‘How frequently does the person express love or affection toward others?’. The POS has acceptable internal consistency (*α* range: 0.40–0.86) and inter‐rater reliability (*r* range: 0.29–0.79; van Loon et al., 2013). The internal consistency of the three domains specifically examined in our study (self‐determination, social inclusion and emotional well‐being) was 0.80, 0.74 and 0.69, respectively. In our own sample, the internal consistency of the three domains was 0.70, 0.69 and 0.58.

#### 2.4.7 Challenging behaviours: Aberrant Behaviour Checklist

The primary staff member reported on challenging behaviours of individuals with intellectual disabilities with the Aberrant Behaviour Checklist (ABC; Aman et al., 1985). The ABC is a widely used scale in clinical practice and research, measuring challenging behaviours in individuals with intellectual disabilities. The instrument includes five subscales: Irritability, Lethargy, Stereotypic Behaviour, Hyperactivity/ Noncompliance and Inappropriate Speech. The ABC consists of 58 items with a 4‐point Likert scale ranging from 0 (not a problem) to 3 (problem is severe in degree) and has a well‐established reliability and validity.

### 2.5 Statistical analyses

We conducted all analyses twice. First, we examined the association of individual staff characteristics with quality of life of the individual, using the scores of the primary informant. This was in the majority of cases the primary staff member of the individuals with intellectual disabilities and challenging behaviours. Second, we examined the characteristics of staff teams in relation to quality of life of the individual. For the team characteristics, we needed scores that took into account the team as a whole and the individual variation on each measure within the team. We could not use mean team scores, since these only reflect an average of high and low scoring team members, but provide no information on the variation around the mean in teams. Mean scores would thus result in loss of valuable information. Alternatively, in order to better discriminate between teams, we calculated percentages of individual staff members within each team who had high scores on each measure of staff characteristics. These high scores on the measures of characteristics were derived from the explanation of the Likert scale scores of the instrument. We considered the following scores as high scores: ‘6’ or higher on the External Control scale of the CDS (6 meaning ‘I agree with this statement’), ‘5’ or higher on the CBSES (5 meaning ‘I agree with this statement’), ‘2’ or higher on the ERCBS (2 meaning ‘Yes, frequent’) and ‘4’ or higher on the SCIBI subscales (4 meaning ‘Highly applicable’). For working experience we calculated the mean years of experience of our sample (*M* = 10.97, rounded up to *M* = 11) and determined a cut‐off score of ‘12 years' to indicate above average as opposed to mean or below average. With regard to educational level, the percentage of staff members who completed a level 3 education (i.e., higher professional education or master) was used. For the team variable sex we used the percentage of male staff members within a team.

Data were analysed using SPSS software (IBM SPSS Statistics for Windows, version 23.0). We checked the dispersion of the data on every staff and team characteristic. When there was limited dispersion (i.e., less than 15% of the participants scored above the determined cut‐off scores), variables were excluded from the subsequent analyses. Additionally, we examined the strength of correlations between variables (i.e., a correlation below 0.3 was considered small, between 0.3 and 0.5 medium and above 0.5 strong; Cohen, 1992), in order to exclude variables in case of strong correlations (i.e., if there was little distinction between variables).

Given the nesting (complete staff teams and individuals with intellectual disabilities and challenging behaviours living together in group homes) and hierarchical structure (staff members within group homes, group homes within organisations) of our data, we conducted multilevel analyses to account for the statistical dependency of the data (Snijders & Bosker, 2012). Multilevel analyses are very suitable for studies which include different levels of aggregation such as our study. Organisation was included as the first level and group home as the second level. First, we conducted the multilevel models for the association between primary staff characteristics and self‐determination, social inclusion, emotional well‐being and quality of life. Then we repeated the analyses with the team characteristics instead of the primary staff characteristics.

For both multilevel analyses we used a Maximum Likelihood model with an unstructured covariance type. The random effects of the models were organisation and group home. We corrected for the possible confounding effects of the level of intellectual disability and challenging behaviours by adding these to the model as covariates. To distinguish significant associations of the staff characteristics with quality of life (all domains and total), we examined the *χ* ^2^_Change_ (based on −2 log‐likelihood and *df*_Change_) with every variable (fixed effect) added to the models. According to the *χ* ^2^ distribution, a *χ* ^2^_Change_ larger than 3.84 was considered significant with *p* < .05 (2‐tailed), and *χ* ^2^_Change_ values larger than 6.63 were considered significant at the level of *p* < .01 (2‐tailed).

The sequence of adding variables to the individual staff models was based on the substantiation in the literature. Variables for which there was quantitative evidence were added first, then variables reported in qualitative studies, and finally variables that were studied in quantitative research but had no or inconclusive quantitative evidence: sex, experience, friendly behaviour, positive emotions to challenging behaviours, hostile behaviour, attributions about external control, self‐efficacy, educational level, self‐reflection, proactive thinking and assertive control. The sequence of variables of the team models was determined by the size of the *χ* ^2^_Change_ values from the primary staff models (i.e., variables with the largest *χ* ^2^_Change_ first).

## 3 RESULTS

### 3.1 Participant characteristics

In total, 25 group homes were included in the study, containing 240 staff members and 152 individuals with intellectual disabilities who all displayed challenging behaviours. The mean team size was 9.6 staff members (range 4–19). The mean number of individuals with intellectual disabilities and challenging behaviours living in a group home was 6.08 (range 3–9). Tables 1 and 2 present characteristics of staff members and of the individuals with intellectual disabilities and challenging behaviours, respectively.

**TABLE 1:** Characteristics of participating staff members (total *N* = 240)

	*n* (%)	Mean (*SD*)	Range	*n* (%) scoring high*
Age		36.6 (11.6)	18–68	
Sex				
Male	83 (34.6%)			
Female	157 (65.4%)			
Working experience (years)		10.9 (9.42)	0.00–43	83 (34.6)
Education				
Level 1: general secondary education	7 (2.9%)			
Level 2: senior secondary vocational education	174 (72.5%)			
Level 3: higher professional education or master	57 (23.8%)			
Causal Dimension Scale (CDS)				
External controllability		5.48 (1.24)	1–9	86 (35.9)
Challenging Behaviour Self‐Efficacy Scale (CBSES)		5.40 (0.75)	2.60–7	184 (76.7)
Emotional Reactions to Challenging Behaviour Scale (ERCBS)				
Positive emotions		1.73 (0.57)	0.23–3	96 (40)
Negative emotions		0.53 (0.26)	0.00–1.53	0
Staff‐Client Interactive Behaviour Inventory (SCIBI)				
Assertive control		2.99 (0.71)	1–4.86	25 (10.4)
Hostile behaviour		2.57 (0.80)	1–5	13 (5.42)
Friendly behaviour		4.16 (0.57)	1–5	186 (77.5)
Support‐seeking behaviour		1.89 (0.72)	1–3.67	0
Proactive thinking		4.01 (0.67)	1–5	176 (73.3)
Self‐reflection		3.19 (0.80)	1–5	49 (20.4)
Critical expressed emotion		1.72 (0.56)	1–3.20	0

a Working experience: 12 years or more; education: level 3; CDS: ‘6’ or higher; CBSES: ‘5’ or higher; ERCBS: ‘2’ or higher; SCIBI: ‘4’ or higher.

**TABLE 2:** Characteristics and outcome measures of the individuals with intellectual disabilities and challenging behaviours in the study (total *N* = 152)

	*n* (%)	Mean (*SD*)	Range
*Characteristics*			
Age*		40.25 (15.23)	18–73
Sex			
Male	90 (59.2)		
Female	62 (40.8)		
Level of intellectual disability			
Mild	26 (17.1)		
Moderate	61 (40.1)		
Severe	38 (25.0)		
Profound	24 (15.8)		
Unknown	3 (2.0)		
Aberrant Behaviour Checklist (ABC)^a^ ^,^ ^b^		48.38 (23.62)	5–130
*Outcome measure*			
Personal Outcome Scale (POS)			
Total quality of life		102.11 (7.92)	80–121
Self‐Determination		14.14 (2.05)	8–18
Social Inclusion		9.54 (2.41)	6–16
Emotional Well‐Being		15.28 (1.99)	8–18

^a^ Missing data of two participants.

^b^ Missing data of one participant.

As is shown in Table 1, the mean scores on the staff variables ‘negative emotions’, ‘support‐seeking behaviour’ and ‘critical expressed emotion’ were relatively low and there was limited dispersion towards the higher end of the scale. Therefore, these variables were not included in the multilevel analyses. The dispersion on the team variable ‘hostile behaviour’ was low with only some clear outliers. Therefore, we also excluded this variable from the team analyses. All other variables were included.

### 3.2 Associations between characteristics of individual staff members and quality of life

The multilevel analyses (Table 3) show that education level and self‐reflection of staff significantly contribute to the emotional well‐being model (*χ* ^2^_Change_(1) = 4.636, and *χ* ^2^_Change_(1) = 6.096, respectively). Higher education and higher self‐reflection of individual staff members were associated with better emotional well‐being of individuals with intellectual disabilities and challenging behaviours (*β* = 3.499, and *β* = 0.516, respectively). No other variables contributed significantly to any of the models.

**TABLE 3:** Multilevel analyses of associations between individual staff characteristics and self‐determination, social inclusion, emotional well‐being and total quality of life of individuals with intellectual disabilities and challenging behaviours

Model 1: Self‐determination			
	−2LogLikelihood (*df*)	*Χ* ^2^_Change_ (*df*_Change_)	*β*
Fixed effect			
Intercept	632.229 (3)		
Sex	632.065 (4)	0.164 (1)	0.158
Experience	631.751 (5)	0.314 (1)	−0.01
Friendly behaviour	630.027 (6)	1.724 (1)	−0.468
Positive emotions to CB	629.461 (7)	0.566 (1)	−0.223
Hostile behaviour	629.250(8)	0.211(1)	0.095
Attribution external control	629.244(9)	0.006 (1)	0.010
Self‐efficacy	628.510 (10)	0.734 (1)	−0.231
Education level	624.918 (11)	3.592 (1)	3.118
Self‐reflection	624.728(12)	0.190 (1)	−0.093
Proactive thinking	624.191 (13)	0.537 (1)	−0.202
Assertive control	623.794 (14)	0.397 (1)	−0.191

Model 2: social inclusion			
	−2LogLikelihood (*df*)	*Χ* ^2^_Change_ (*df*_Change_)	*β*
Fixed effect			
Intercept	674.674 (3)		
Sex	672.836 (4)	1.838 (1)	−0.589
Experience	672.419(5)	0.417 (1)	−0.013
Friendly behaviour	671.264 (6)	1.155 (1)	−0.440
Positive emotions to CB	671.260 (7)	0.004 (1)	−0.024
Hostile behaviour	671.195 (8)	0.065 (1)	0.062
Attribution external control	671.195 (9)	0 (1)	0.001
Self‐efficacy	670.739 (10)	0.456 (1)	0.215
Education level	670.236 (11)	0.503 (1)	0.263
Self‐reflection	669.723(12)	0.513 (1)	−0.180
Proactive thinking	669.227 (13)	0.496 (1)	−0.223
Assertive control	665.667 (14)	3.56 (1)	−.656

Model 3: emotional well‐being			
	−2LogLikelihood (*df*)	*Χ* ^2^_Change_ (*df*_Change_)	*β*
Fixed effect			
Intercept	632.928 (3)		
Sex	632.928 (4)	0 (1)	0.007
Experience	631.496 (5)	1.432 (1)	−0.022
Friendly behaviour	631.490 (6)	0.006 (1)	0.029
Positive emotions to CB	629.229 (7)	2.261 (1)	0.454
Hostile behaviour	628.290 (8)	0.939 (1)	−0.194
Attribution external control	625.897 (9)	2.393 (1)	0.199
Self‐efficacy	624.993 (10)	0.904 (1)	0.254
Education level	620.357 (11)	4.636* (1)	3.499
Self‐reflection	614.261 (12)	6.096* (1)	0.516
Proactive thinking	614.019 (13)	0.242 (1)	−0.130
Assertive control	612.086 (14)	1.933 (1)	−0.411

Model 4: total quality of life score			
	−2LogLikelihood (*df*)	*Χ* ^2^_Change_ (*df*_Change_)	*β*
Fixed effect			
Intercept	1021.594 (3)		
Sex	1020.146 (4)	1.448 (1)	1.688
Experience	1019.253 (5)	0.893 (1)	−0.060
Friendly behaviour	1018.696 (6)	0.557 (1)	0.940
Positive emotions to CB	1018.408 (7)	0.288 (1)	0.562
Hostile behaviour	1015.820 (8)	2.588 (1)	−1.201
Attribution external control	1012.522 (9)	3.298 (1)	0.770
Self‐efficacy	1011.601 (10)	0.921 (1)	−0.896
Education level	1008.281 (11)	3.320 (1)	8.022
Self‐reflection	1005.585 (12)	2.696 (1)	−1.210
Proactive thinking	1003.506 (13)	2.079 (1)	−1.335
Assertive control	1002.984 (14)	0.522 (1)	−0.744

*Note*: CB, challenging behaviours; *Χ ^2^_Change_ is significant at *p* < .05 (2‐tailed).

### 3.3 Associations between characteristics of staff teams and quality of life

Our multi‐level analyses, presented in Table 4, demonstrate that education level and the staff‐individual ratio contributed significantly to the model of self‐determination (*χ* ^2^_Change_(1) = 5.312 and *χ* ^2^_Change_(1) = 10.334, respectively); a higher percentage of highly educated staff members within a team was associated with more self‐determination of individuals with intellectual disabilities and challenging behaviours, and a higher staff‐individual ratio was associated with less self‐determination (*β* = 0.037 and *β* = −5.072, respectively). Further, education level and staff‐individual ratio contributed significantly to the social inclusion model (*χ* ^2^_Change_(1) = 4.615 and *χ* ^2^_Change_(1) = 8.473, respectively); a higher percentage of highly educated staff members in a team was associated with more social inclusion of individuals with intellectual disabilities and challenging behaviours (*β* = 0.038). A higher staff‐individual ratio was associated with less social inclusion (*β* = −4.943). Furthermore, staff self‐efficacy and friendly behaviour significantly contributed to the emotional well‐being model (*χ* ^2^_Change_(1) = 4.208 and *χ* ^2^_Change_(1) = 6.253, respectively). More staff members in a team reporting high self‐efficacy and more staff members in a team reporting much friendly behaviour were associated with better emotional well‐being of individuals with intellectual disabilities and challenging behaviours (*β* = 0.023 and *β* = 0.033, respectively). Finally, staff education level contributed significantly to the model of total quality of life scores (*χ* ^2^_Change_(1) = 12.234). A higher percentage of highly educated staff members within a team was associated with better quality of life of individuals with intellectual disabilities and challenging behaviours (*β* = 0.211).

**TABLE 4:** Multilevel analyses of associations between staff team characteristics and self‐determination, social inclusion, emotional well‐being, and total quality of life of individuals with intellectual disabilities and challenging behaviours

Model 5: Self‐determination			
	−2LogLikelihood (*df*)	*Χ* ^2^_Change_ (*df*_Change_)	*β*
Fixed effect			
Intercept	616.861 (3)		
Education level	611.549 (4)	5.312* (1)	0.037
Friendly behaviour	609.927 (5)	1.622 (1)	−0.018
Self‐efficacy	609.664 (6)	0.263 (1)	−0.007
Positive emotions to CB	609.659 (7)	0.005 (1)	−0.001
Proactive thinking	609.639 (8)	0.020 (1)	0.003
Assertive control	609.465 (9)	0.174 (1)	−0.008
Experience	609.455 (10)	0.010 (1)	−0.002
Self‐reflection	608.309 (11)	1.146 (1)	−0.031
Sex	606.374 (12)	1.935 (1)	−0.016
Attribution external control	606.301 (13)	0.073 (1)	0.004
Staff‐individual ratio	595.967 (14)	10.334** (1)	−5.072

Model 6: social inclusion			
	−2LogLikelihood (*df*)	*Χ* ^2^_Change_ (*df*_Change_)	*β*
Fixed effect			
Intercept	669.164 (3)		
Assertive control	669.072 (4)	0.092 (1)	0.007
Sex	668.914 (5)	0.158 (1)	−0.005
Friendly behaviour	667.594 (6)	1.32 (1)	−0.023
Self‐reflection	667.319 (7)	0.275 (1)	−0.016
Education level	662.704 (8)	4.615* (1)	0.038
Proactive thinking	660.360 (9)	2.344 (1)	0.036
Self‐efficacy	660.342 (10)	0.018 (1)	0.002
Experience	659.966 (11)	0.376 (1)	−0.011
Positive emotions to CB	659.553 (12)	0.413 (1)	0.012
Attribution external control	656.543 (13)	3.01 (1)	−0.026
Staff‐individual ratio	648.070 (14)	8.473** (1)	−4.943

Model 7: emotional well‐being			
	−2LogLikelihood (*df*)	*Χ* ^2^_Change_ (*df*_Change_)	*β*
Fixed effect			
Intercept	632.051 (3)		
Self‐reflection	631.898 (4)	0.153 (1)	0.009
Education level	629.186 (5)	2.712 (1)	0.021
Attribution external control	629.163 (6)	0.023 (1)	−0.002
Positive emotions to CB	625.974 (7)	3.189 (1)	0.022
Assertive control	625.937 (8)	0.037 (1)	0.003
Experience	625.937 (9)	0 (1)	0.001
Self‐efficacy	621.729 (10)	4.208* (1)	0.023
Proactive thinking	621.057 (11)	0.672 (1)	−0.013
Friendly behaviour	614.804 (12)	6.253* (1)	0.033
Sex	614.135 (13)	0.669 (1)	−0.007
Staff‐individual ratio	614.132 (14)	0.003 (1)	−0.061

Model 8: total quality of life score			
	−2LogLikelihood (*df*)	*Χ* ^2^_Change_ (*df*_Change_)	*β*
Fixed effect			
Intercept	1006.485 (3)		
Education level	994.251 (4)	12.234** (1)	0.211
Attribution external control	993.525 (5)	0.726 (1)	−0.040
Self‐reflection	993.509 (6)	0.016 (1)	−0.011
Proactive thinking	993.301 (7)	0.208 (1)	0.029
Sex	991.952 (8)	1.349 (1)	−0.042
Self‐efficacy	991.443 (9)	0.509 (1)	0.033
Experience	991.303 (10)	0.140 (1)	−0.006
Friendly behaviour	990.095 (11)	1.208 (1)	−0.075
Assertive control	990.056 (12)	0.039 (1)	−0.013
Positive emotions to CB	989.940 (13)	0.116 (1)	0.048
Staff‐individual ratio	986.610 (14)	3.330 (1)	−12.431

*Note*: CB, challenging behaviours; *Χ ^2^_Change_ is significant at *p* < .05 (2‐tailed), **Χ ^2^_Change_ is significant at *p* < .01 (2‐tailed).

## 4 DISCUSSION

In this study, we investigated the association of characteristics of both individual staff and staff teams with quality of life of individuals with intellectual disabilities and challenging behaviours. We found that more self‐reflection in individual staff members and teams in which more staff members reported higher levels of friendly behaviour and self‐efficacy were associated with better emotional well‐being of the individuals under their care. These findings empirically corroborate earlier qualitative studies (Petry et al., 2007; Webb et al., 2020; Windley & Chapman, 2010) in which individuals with intellectual disabilities, parents and staff members perceived friendly staff behaviours and staff self‐reflection as important characteristics to increase the quality of life of the individuals concerned. Additionally, our study is the first to find a direct relation of staff self‐reflection and friendly staff behaviour with quality of life. This adds to the body of research that found self‐reflection of staff was associated with lower hostile behaviour of staff (Willems et al., 2016), which in turn was related to lower quality of life (Griffith et al., 2013). However, to unravel the direction of the associations in order to understand possible causality, more research applying longitudinal designs is necessary.

As expected, a higher educational level of individual staff members was positively associated with better emotional well‐being of the individuals with intellectual disabilities and challenging behaviours, and more staff members within a team with a higher education level was associated with better self‐determination, social inclusion and quality of life of the individuals. The findings regarding individual staff confirm results of earlier studies that found that higher education in individual staff members was related with better quality of life of the individuals under their care (Mansell et al., 2008; Thomas et al., 1978). However, our study indicates that the proportion of higher educated staff members within a team is somewhat differently related to quality of life than higher education in individual staff. The latter was only related to emotional well‐being, whereas the first was more broadly related to quality of life (i.e., self‐determination, social inclusion, and total quality of life). These findings demonstrate the value for future research to take complete staff teams into account when examining staff factors and quality of life.

Contrary to our expectations, higher staff‐individual ratios (i.e., more staff members working per individual with intellectual disabilities) were associated with lower self‐determination and less social inclusion of the individuals with intellectual disabilities and challenging behaviours. Since we corrected for the level of intellectual disability and degree of challenging behaviours, these results cannot be attributed to these factors. However, we did not take into account other factors that may be related to higher staff‐individual ratios and possibly lower quality of life, such as physical or communication problems of the individuals or a diagnosis of autism spectrum disorder (Buntinx & Schalock, 2010; Petry et al., 2007). These factors may relate to lower quality of life as has been reported in a study of Beadle‐Brown et al. (2016), in which they examined quality of life of individuals with severe intellectual disabilities and complex needs (i.e., comorbid problems such as challenging behaviours, autism, physical disabilities and communication problems) living in community‐based services with high staff‐individual ratios. They found that the quality of life of this population was relatively poor. This supports our assumption that the association of staff‐individual ratio with quality of life might be moderated by characteristics of the individuals with intellectual disabilities in settings with high staff‐individual ratios. Therefore, we would recommend future studies that examine the relation between staff‐individual ratio and quality of life to better investigate the characteristics of the individuals with intellectual disabilities in the study (e.g., comorbid problems).

### 4.1 Strengths and limitations

With this paper, we add to the scarce quantitative studies examining staff and team of staff characteristics that may be associated with quality of life of individuals with intellectual disabilities and challenging behaviours. Never before was total quality of life taken into account, or did studies differentiate between primary staff characteristics and team of staff characteristics. However, our study has limitations. Unfortunately, we could not examine the association between negative staff factors and quality of life of individuals with intellectual disabilities and challenging behaviours. Our data on negative staff emotions in response to challenging behaviours, staff critical expressed emotions and hostile staff behaviour showed overall low scores and little dispersion and could therefore not be included in the analyses. Although the scores on these variables were comparable to other studies that used the same instruments (Jones & Hastings, 2003; Willems et al., 2014, 2016), it is questionable if these negative factors did indeed not occur in our sample or if the staff members answered socially desirable, which is a known concern (Lambrechts et al., 2010). Some staff members may experience a mismatch between their initial response to challenging behaviour (i.e., fight or flight) and their belief of what their response should be as a professional (i.e., reacting according to the formal treatment plan; Feldman et al., 2004; Jahoda & Wanless, 2005). It would be valuable for research and clinical practice to gain insight on how observable behavioural responses of staff on challenging behaviours correspond with reported emotional reactions to challenging behaviours, and how these actual behaviours are associated with quality of life of the individuals. Therefore, future research should use observational measures to assess such staff behaviours.

In interpreting our findings, it should be acknowledged that we had to use proxy measures for assessing quality of life (i.e., by interviewing the primary staff member), as we included individuals of all levels of intellectual disabilities. Even though the POS manual reports no significant differences in outcomes between self‐report or reports of others (van Loon et al., 2013), social desirable answering of the informant could have affected our results (Sexton et al., 2016; Van Hecke et al., 2018). Moreover, a limitation was that we used the same source (i.e., [primary] staff members) for the measurement of our independent variables (i.e., individual staff and staff team characteristics) and dependent variables (i.e., quality of life). Therefore, we recommend future studies to take more proxies into account for different perspectives and greater reliability. Another limitation is that we did not include all potentially important staff and team of staff characteristics in our study. For example, it would be interesting to examine the effect of (PBS) staff training, organisational culture, staff turnover, job satisfaction of staff, heterogeneity within staff teams, the relationship of individuals with intellectual disabilities with their family members or the relationships between staff members into account (Bigby & Beadle‐Brown, 2018). Furthermore, it is important to improve our insight into the associations of various individual characteristics such as the presence of comorbid disorders, physical well‐being, and housing situation with the quality of life of individuals with intellectual disabilities and challenging behaviours. Future studies should include these factors when examining relations between characteristics of (teams of) staff individual characteristics with quality of life of individuals with intellectual disabilities and challenging behaviours.

### 4.2 Clinical implications

The results of our study provide insights with relevance for clinical practice. First, we would like to highlight the need to educate staff members in how their attitude may be related to the quality of life of the individuals they care for. Our study also showed a positive association between higher educational level of staff with several quality of life domains of individuals with intellectual disabilities and challenging behaviours. Unfortunately, many staff members are often undereducated and not aware about their possible impact on quality of life of the individuals under their care (McConkey & Collins, 2010). Second, although our findings showed correlational (and no causal) relations between staff characteristics and quality of life, they may point to the importance of training staff to increase their confidence, self‐reflection skills and friendly behaviour. These staff characteristics are not only associated with better emotional well‐being of individuals with intellectual disabilities and challening behaviours, but also to decreased stress and burnout (Zijlmans et al., 2015), which are major problems in the clinical field of intellectual disabilities (Finkelstein et al., 2018). Finally, our findings suggest that increased staff‐individual ratios might not improve quality of life. The characteristics of staff teams appear to have more positive associations with quality of life than the number of staff members.

## 5 CONCLUSIONS

Our study was the first to take both individual staff and staff team characteristics into account to examine their relation with quality of life of individuals with intellectual disabilities and challenging behaviours. We found that higher educational level and self‐reflection of individual staff members were related to better emotional well‐being, and, in staff teams, that higher educational level, friendly behaviour and self‐efficacy were associated with higher quality of life (i.e., self‐determination, social inclusion, emotional well‐being, and total quality of life). The associations between higher staff‐individual ratios and lower self‐determination and social inclusion were unexpected. Remarkably, staff team characteristics showed different associations with quality of life than individual staff characteristics. This advocates for future research to include complete staff teams when examining quality of life of individuals with intellectual disabilities and challenging behaviours. Moreover, longitudinal studies, observational measures and specific documentation of the characteristics of the individuals with intellectual disabilities will contribute to our knowledge on how staff characteristics and behaviour are related to quality of life of individuals with intellectual disabilities and challenging behaviours.

## FUNDING INFORMATION

This work was supported and funded by the Dutch Research Council (NWO; grant number 432‐13‐809).

## CONFLICT OF INTEREST

The authors declare no conflict of interest.

## Data Availability

The data of the current study are available on reasonable request from the corresponding author. The data are not publicly available due to privacy or ethical restrictions.
